# Nitrogen Removal over Nitrite by Aeration Control in Aerobic Granular Sludge Sequencing Batch Reactors

**DOI:** 10.3390/ijerph110706955

**Published:** 2014-07-08

**Authors:** Samuel Lochmatter, Julien Maillard, Christof Holliger

**Affiliations:** Laboratory for Environmental Biotechnology, School of Architecture, Civil and Environmental Engineering, Ecole Polytechnique Fédérale de Lausanne (EPFL), 1015 Lausanne, Switzerland; E-Mails: samuel.lochmatter@epfl.ch (S.L.); julien.maillard@epfl.ch (J.M.)

**Keywords:** aeration control, aerobic granular sludge, nitritation, nitrite pathway, sequencing batch reactor

## Abstract

This study investigated the potential of aeration control for the achievement of N-removal over nitrite with aerobic granular sludge in sequencing batch reactors. N-removal over nitrite requires less COD, which is particularly interesting if COD is the limiting parameter for nutrient removal. The nutrient removal performances for COD, N and P have been analyzed as well as the concentration of nitrite-oxidizing bacteria in the granular sludge. Aeration phase length control combined with intermittent aeration or alternate high-low DO, has proven to be an efficient way to reduce the nitrite-oxidizing bacteria population and hence achieve N-removal over nitrite. N-removal efficiencies of up to 95% were achieved for an influent wastewater with COD:N:P ratios of 20:2.5:1. The total N-removal rate was 0.18 kgN·m^−3^·d^−1^. With N-removal over nitrate the N-removal was only 74%. At 20 °C, the nitrite-oxidizing bacteria concentration decreased by over 95% in 60 days and it was possible to switch from N-removal over nitrite to N-removal over nitrate and back again. At 15 °C, the nitrite-oxidizing bacteria concentration decreased too but less, and nitrite oxidation could not be completely suppressed. However, the combination of aeration phase length control and high-low DO was also at 15 °C successful to maintain the nitrite pathway despite the fact that the maximum growth rate of nitrite-oxidizing bacteria at temperatures below 20 °C is in general higher than the one of ammonium-oxidizing bacteria.

## 1. Introduction

Biological wastewater treatment by aerobic granular sludge sequencing batch reactor (AGS-SBR) technology offers the possibility to remove carbon (hereafter referred to as chemical oxygen demand, COD), nitrogen (N) and phosphorus (P) in one single reactor [1,2,3]. Nevertheless, the simultaneous removal of nitrogen and phosphorus is not always successful [4,5]. Denitrifying bacteria and polyphosphate-accumulating organisms (PAO) are both heterotrophic, and PAO as well as some of the former group are able to take up carbon under anaerobic conditions and store it intracellularly until a suitable electron acceptor is available in order to use the stored carbon for growth. In combined denitrification and enhanced biological phosphorus removal (EBPR) systems, COD availability is often the limiting parameter [6,7], and PAO and denitrifiers are in direct competition for the available COD. Both processes have been reported to be potentially disturbed by this competition. Several studies reported an accumulation of nitrate due to a lack of COD, whereas the P-removal efficiency remained quite high [3,8,9]. On the other hand, it has also been reported that the presence of nitrate (NO_3_^−^) and/or nitrite (NO_2_^−^) can disturb P-removal in sequencing batch mode [10,11]. Presence of nitrate and/or nitrite (nitrate and nitrite = NO_x_^−^) at the end of the sequencing batch cycle leads to anoxic conditions in the subsequent feeding phase. Under such conditions, part of the COD is consumed by denitrifying bacteria, leaving less carbon for PAO to be stored in the form of polyhydroxyalkanoates during the anaerobic phase resulting in lower P-removal overall [11].

In wastewater treatment plants insufficient denitrification is sometimes improved by the addition of an external carbon source, for example ethanol, whereas P-removal can be realized by a chemical pre- or post-treatment. Nevertheless, these solutions are costly and lead to a higher excess sludge production. Hence, solutions intending to reduce COD requirements are needed. A well-known approach to reduce COD requirements is N-removal over nitrite. Ammonium is only oxidized to nitrite, but not to nitrate, and then denitrified. From stoichiometric equations with methanol as carbon source, it can be derived that 25% less oxygen and 40% less COD is required to transform ammonium (NH_4_^+^) into nitrogen gas (N_2_) over nitrite compared to conventional N-removal over nitrate. Other advantages are a lower biomass production and a higher denitrification rate [12]. To achieve the nitrite pathway, conditions have to be created such that the second nitrification step from nitrite to nitrate is not occurring. This means, that strategies have to be implemented, which are unfavorable for nitrite-oxidizing bacteria (NOB) but not for ammonium-oxidizing bacteria (AOB). Such strategies for the negative selection of NOB are moreover of rising interest, since they are also needed for the so-called partial nitritation/anammox (PN/A) process, one of the emerging technologies in biological wastewater treatment [13]. 

One of the first applied examples of a selective elimination of NOB has been the so-called Single reactor High activity Ammonia Removal over Nitrite (SHARON process; Hellinga *et al.* [14]). At temperatures >25 °C, AOB have a higher maximum specific growth rate than NOB [14]. Hence, by reducing the sludge retention time (SRT), NOB can be washed out. This strategy works only for elevated temperatures, and although large variations of growth rates for nitrifiers have been reported in literature [15], it is widely accepted that at temperatures <20 °C, the maximum specific growth rate of NOB is higher than the growth rate of AOB. Hence, for temperatures <20 °C strategies acting directly on the growth rate of and suitable growth conditions for NOB are required. Several chemical substances such as free ammonia (FA) and free nitrous acid (FNA) [16,17], chlorate [18] or formic acid [19] have been shown to selectively inhibit NOB. Besides chemical inhibitors, the competition for oxygen has been used as a parameter to achieve N-removal over nitrite. AOB have a lower oxygen half-saturation coefficient than NOB [20,21], which means that NOB are more sensitive to low oxygen concentrations. Several studies confirmed the possibility to achieve the nitrite pathway with low dissolved oxygen (DO) concentrations [22,23,24]. Beside low DO, aeration phase length control [25,26] and intermittent aeration [27,28] have been investigated with sequencing batch reactors (SBR) for the achievement or maintenance of the nitrite pathway. With aeration phase length control, the aeration has been stopped upon completion of ammonium oxidation. Lemaire *et al.* [26] have advanced the following mechanism to explain the elimination of NOB by this strategy: nitrite oxidation starts and finishes naturally after ammonium oxidation. If the aeration is stopped prior to or right at the moment of complete ammonium oxidation, no extra time is left for the completion of the nitrite oxidation by NOB. It gradually reduces the energy supply for NOB and therefore its growth. Over the cycles it leads to a gradual decrease of NOB. In the study of Lemaire *et al.* [26], wastewater has been fed after stopping aeration to supply COD for post-denitrification. In Blackburne *et al.* [25] formic acid has been used to reduce the NOB population at the beginning of the study, where the main mechanism of nitrite elimination was pre-denitrification during the feeding phase of the subsequent cycle. The temperature was in both studies around 20 °C. Fux *et al.* [28] have implemented an automated intermittent aeration strategy based on the oxidation–reduction potential (ORP). They achieved the nitrite pathway in a SBR operated at 30–32 °C and with ethanol supply during the non-aerated phases to enhance denitrification. Li *et al.* [27] have maintained partial nitrification with intermittent aeration in a SBR operated at 20 °C in a long-term study. NOB have been eliminated by free ammonia inhibition and high temperature. Similar strategies based on controlled intermittent aeration have recently been implemented for continuous reactors and mainstream wastewater. Ge *et al.* [29] applied intermittent aeration and step-feed to a plug-flow reactor at 20 °C. Regmi *et al.* [30] developed an aeration control strategy based on *in situ* measurement of NH_4_^+^, NO_3_^−^ and NO_2_^−^, which has been successfully tested for the NOB out-selection at 25 °C in a continuously stirred tank reactor. Very limited research has been done so far on achieving or maintaining the nitrite pathway in SBR at temperatures below 20 °C. Stable partial nitrification at temperatures fluctuating between 12 and 27 °C has been reported in a SBR initially operated at 27 °C [31], as well as in a one-stage PN/A SBR were the temperature has been gradually lowered from 25 to 12 °C [32]. Yang *et al.* [33] maintained N-removal over nitrite with automated aeration control and step-feeding, while gradually decreasing the temperature from 22 to 12 °C. 

The aim of this study was to achieve and maintain the nitrite pathway at temperatures of 20 °C and 15 °C by aeration control in a SBR with aerobic granular sludge (AGS) for biological nutrient removal. A combination of aeration phase length control and DO variation was tested to achieve N-removal over nitrite, without using chemical NOB inhibitors and without additional carbon supply after the feeding phase. The COD for denitrification came from the carbon stored intracellularly by certain bacteria during the anaerobic feeding phase. A matter of particular interest was the time necessary to achieve the nitrite pathway, because the switch from the nitrate to the nitrite pathway with aeration control has been reported to be a very slow process of 100–300 days [25,26].

## 2. Materials and Methods

### 2.1. Technical Setup

Two identical bubble column reactors with diameters of 5.2 cm and working volumes of 2.4 L were used in this study as described in Ebrahimi *et al* [34]. Granular sludge was cultivated in one of the two reactors according to the strategy developed in Lochmatter *et al* [35]. The flocculent sludge used for inoculation was provided by the wastewater treatment plant (WWTP) Thunersee (Switzerland) and had a sludge volume index of about 90 mL·g^−1^. The activated sludge-based WWTP Thunersee treats N and P biologically in a continuous anaerobic-anoxic-aerobic process. 

The SBR cycle was composed of four steps: an anaerobic feeding phase of 60 min, a starvation phase of 100–150 min according to the aeration strategies described below, 3–5 min settling, and 5 min for withdrawal. It resulted in a total SBR cycle time of maximum 220 min. The water exchange ratio per cycle was 50%. The pH was measured with a glass probe (Mettler Toledo, Nänikon, Switzerland) and controlled between 7.0 and 7.3 by the injection of 1 mol·L^−1^ NaOH and HCl solutions.

The DO concentration was measured by an Ingold membrane electrode (Mettler Toledo) connected to a signal amplifier (Endress + Hauser, Reinach, Switzerland). For an easier regulation of the DO concentration the headspace gas was recirculated as suggested by Mosquera-Corral *et al.* [36], with air or N_2_ supply if the measured DO was outside the range of ±3% to the set point. The air and N_2_ supply was regulated by proportional-integral-derivative (PID) controlled mass flow controllers (MFC). The total gas flow was kept constant at 3.6 L·min^−1^ during the aerated phases, independent of the DO set point. 

The influent wastewater consisted of a mixture of two synthetic media and tap water adapted from de Kreuk *et al.* [2]. Aliquots of 130 mL of each medium were diluted with 940 mL of tap water. Medium A was composed of 4.28 g·L^−1^ of sodium acetate trihydrate, 3.51 g·L^−1^ of sodium propionate, 0.89 g·L^−1^ of MgSO_4_·7H_2_O, and 0.36 g·L^−1^ of KCl, medium B of 1.89 g·L^−1^ of NH_4_Cl, 0.73 g·L^−1^ of K_2_HPO_4_, 0.23 g·L^−1^ of KH_2_PO_4_, and 5 mL·L^−1^ of a trace element solution as described by Vishniac and Santer [37]. This composition resulted in concentrations of about 400 mgCOD·L^−1^, 50 mgN-NH_4_^+^·L^−1^, and 20 mgP-PO_4_^3−^·L^−1^, with an acetate-to-propionate ratio of 1:1 according to COD equivalents.

### 2.2. Experimental Schedule and Aeration Strategies

**Figure 1:**
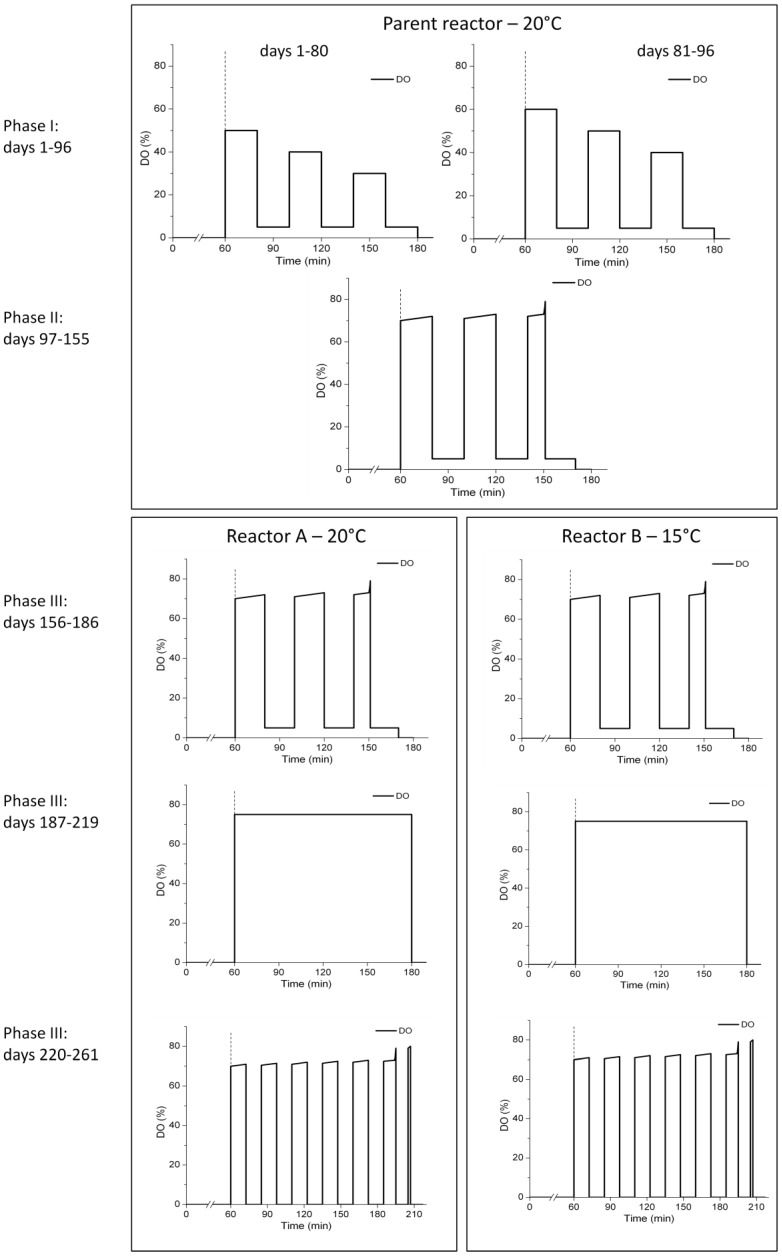
Schematic representation of the aeration strategies during the five experimental phases.

The study was divided in five phases. Phase I from day 1 to 96: the granular sludge was produced from flocculent activated sludge in a parent reactor operated with alternate high-low DO periods (Figure 1). The two hours of aeration were split into 3 times 40 min of alternate high-low DO, with high and low DO periods of 20 min each. From day 1 to 80, the set points of the three high DO periods were at 50%, 40% and 30% of DO saturation, respectively and the low DO set point at 5%. At day 81, the set points of the high DO periods were increased by 10% to 60%, 50% and 40%, respectively. 

In Phase II from day 97 to 155, the reactor was aerated with 3.6 L·min^−1^ of air during the high DO periods, without defined set points. However, the last high DO period was stopped when ammonium oxidation was upon completion, based on the DO signal. The temperature was maintained at 20 °C during Phases I and II.

For Phase III that lasted from day 156 to 186, the granular sludge of the parent reactor was distributed over two reactors, one operated at 20 °C (reactor A) and the other at 15 °C (reactor B) (Figure 1). The aeration strategy was the same as in Phase II, alternate high-low DO with uncontrolled full aeration during the high DO periods and with aeration phase length control (Figure 1). In Phase IV from day 187 to 219, the reactors were fully aerated during 2 h without any low DO periods (Figure 1). Finally, in Phase V from day 220 to 261, the reactors were operated with intermittent aeration and aeration phase length control (Figure 1). Aeration pulses of 12.5 min were followed by idle periods of 12.5 min without aeration or mixing. A maximum of six aeration pulses were carried out. If all ammonium was consumed before the end of the sixth aeration pulse, the aeration was stopped earlier. After the aeration stop, another period of 10.5 min without aeration was added, followed by a short aeration pulse of 2 min. The 2 min aeration pulse served to mix the granular sludge once again before final settling. 

### 2.3. Analytical Methods

Dissolved chemical compounds were analyzed as described in Gonzalez-Gil and Holliger [38]: Concentrations of organic acids were measured by high performance liquid chromatography (HPLC) (Jasco Co-2060 Plus, refraction index detector; Brechbühler AG, Schlieren, Switzerland) with organic acid ion-exclusion column ORH-801 (Transgenomic, Glasgow, United Kingdom). Inorganic cations and anions such as ammonium (NH_4_^+^), nitrate (NO_3_^−^), nitrite (NO_2_^−^), and orthophosphate (PO_4_^3−^) were measured by ion-chromatography (anions: ICS-90, IonPacAS14A column; cations: ICS-3000A, IonPacCS16 column) with electrical conductivity detection (Dionex, Olten, Switzerland). The total (TSS) and volatile suspended solids (VSS) were analyzed as described in Lochmatter *et al.* [9].

### 2.4. Nitrification Batch Tests

Nitrification batch tests were carried out in a 1 L glass beaker with 0.5 L working volume and about 2 gVSS granular sludge. The sludge was taken from the SBR at the end of the aeration phase. An aliquot of an NH_4_Cl stock solution (140 mgN·L^−1^) resulting in a final concentration of 17 mgN·L^−1^ was added. A porous diffuser was used for aeration, with an airflow high enough to completely mix the bulk liquid. The test was carried out during 2 h with liquid sampling every 20 min for ammonium, nitrite, and nitrate analysis.

### 2.5. Quantification of Nitrite-Oxidizing Bacteria

NOB were analyzed by quantitative PCR (qPCR) targeting specifically the 16S rRNA gene of *Nitrospira* since pyrosequencing analysis has previously shown that *Nitrospira* was the main NOB in our aerobic granular sludge [39]. A reference plasmid named pNOB was used as standard. This plasmid was obtained by cloning a 151-bp fragment of the *Nitrospira* 16S rRNA gene amplified from total DNA obtained from granular sludge with the primers NTSPA-D16S-F (5′-CCTGCTTTCAGTTG CTACCG-3′) and NTSPA-D16S-R (5′-GTTTGCAGCGCTTTGTACCG-3′) taken from Dionisi *et al.* [40]. The PCR reaction mix contained: 66.5 µL of ddH_2_O, 10 µL of 10 × PCR buffer S (Peqlab, Axonlab, Baden, Switzerland), 3 µL of dNTPs at 2.5 mM, 5 µL of 10 µM primer and 0.5 µL of Taq polymerase at 5 U·µL^−1^ (Peqlab). One µL of DNA at 1 ng·µL^−1^ was added as template. The PCR program was designed as follows: 5 min of initial denaturation at 95 °C, 30 cycles of amplification with each cycle including 45 s denaturation at 95 °C, 45 s of primer annealing at 52 °C, and 60 s of elongation at 72 °C; a 5 min step of final elongation at 72 °C was added at the end. The PCR product was purified with the PCR purification Kit (Qiagen, Hombrechtikon, Switzerland) and ligated into the vector pGEM-T Easy (Promega, Dübendorf, Switzerland) following the manufacturer’s instructions. The resulting plasmid was transformed in CaCl_2_-competent *E. coli* DH5α cells using standard heat shock protocol. Transformants were selected by colony PCR and verified by sequencing using in-house facility as described previously in Rupakula *et al.* [41]. A positive transformant harboring pNOB was cultivated for plasmid extraction using the Qiaquick Miniprep Kit (Qiagen). One µg of plasmid DNA was linearized by digestion with the restriction enzyme ScaI (Promega), dephosphorylated by shrimp alkaline phosphatase (TaKaRa, Labegene, Châtel-St-Denis, Switzerland), and finally purified using the PCR purification kit (Qiagen). DNA concentration was measured with a NanoDrop ND-1000 apparatus. Standards for qPCR were prepared by serial dilution from 1.15 × 10^7^ to 10^1^ plasmid copies μL^−1^. 

Runs of qPCR consisted of the standard dilution series and samples in triplicates. The 10 μL qPCR reaction mixture was composed of 5 μL of KAPA SYBR^®^ FAST Universal 2 × qPCR Master Mix, 0.2 μL of primers NTSPA-D16S-F and -R (runs 1–5) at 10 μM, 2.1 μL of sterile water and 2.5 μL of DNA template. The program consisted of 15 min initial denaturation at 94 °C, followed by 40 cycles of 30 s denaturation at 94 °C, 20 s primer annealing at 60 °C, 30 s elongation at 72 °C, and 15 s fluorescence acquisition at 80 °C. A melt curve ranging from 72 to 99 °C was added at the end for quality assessment. The reaction was run in the RotorGene RG3000 machine (Qiagen, Hombrechtikon, Switzerland).

Data were analyzed using the RotorGene 6 software with a fluorescence threshold fixed at 0.25. The copy number (cn) of *Nitrospira* 16S rRNA genes was calculated for each sample using Equation (1) from the average cycle threshold (CT) value (with standard deviation kept below 3% of the average), and the parameters M and B calculated from the run-specific standards dilution series. These parameters along with reaction efficiency and R^2^ value of the linear regression of the standards are given in the Supplementary Data (Table S1):

cn = 10^[(CT − B)/M]^ (1)

### 2.6. Nitrous Oxide Tests

Dissolved concentrations and gaseous emissions of nitrous oxide (N_2_O) were investigated in a separate study. Granular sludge with N-removal over nitrite and nitrate, respectively, was maintained in two SBRs. A liquid phase microsensor (Unisense, Aarhus, Denmark) was used for the continuous measurement of N_2_O in the bulk liquid. The probe was calibrated with nitrogen gas and a N_2_O calibration gas at a concentration of 200 ppm. For both sludge types, N_2_O was measured with two different aeration strategies: constant DO of 30% and intermittent aeration, and with two different influent COD concentrations: 400 and 600 mgCOD·L^−1^ (Table 1).

**Table 1:** Tested conditions for dissolved N_2_O concentrations and N_2_O emissions.

Run	N-Removal over	Aeration Strategy	COD Concentration
No.			(mgCOD·L^−1^)
1	NO_2_^−^	Constant DO 30%	400
2	NO_2_^−^	Constant DO 30%	600
3	NO_2_^−^	Intermittent aeration	400
4	NO_2_^−^	Intermittent aeration	600
5	NO_3_^−^	Constant DO 30%	400
6	NO_3_^−^	Constant DO 30%	600
7	NO_3_^−^	Intermittent aeration	400
8	NO_3_^−^	Intermittent aeration	600

The microsensor gave stable results also for N_2_O concentrations in the gas phase. Eighteen grab samples were analyzed by gas chromatography (HP 5890 series II, RTX-502.2 column, electron capture detector) to compare with the microsensor measurements (Supplementary Figure S1). The same conditions were tested with the microsensor installed in the head-space zone of the SBR reactor to measure N_2_O emissions. The head-space gas recirculation was disconnected during the N_2_O study. All runs were measured over 1 day (8 cycles). 

## 3. Results

### 3.1. Strategy for Aeration Phase Length Control

**Figure 2:**
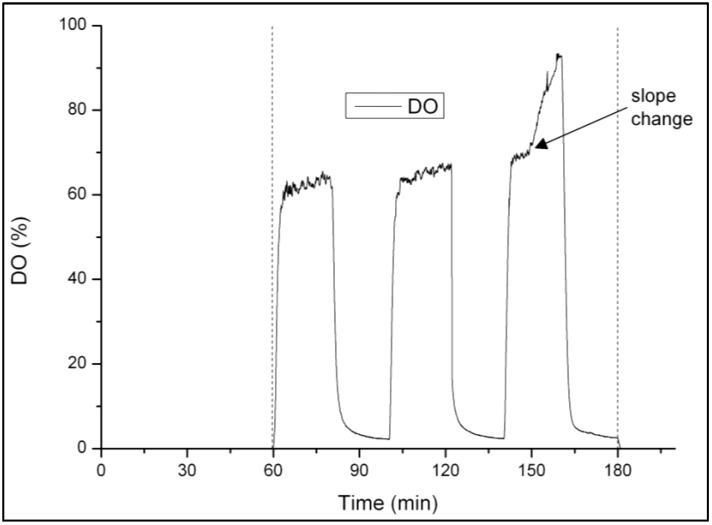
Illustration of a DO profile during a SBR cycle with alternate high-low DO periods with full aeration during the high DO periods. The slope change of DO towards the end of nitrification occurring during the third high DO period is indicated.

The automated aeration phase length control was implemented based on the DO signal, with the aim to stop aeration once ammonium oxidation was upon completion. It was applied between days 97–186 (Phases II and III), and days 220–261 (Phase V). The oxygen uptake rate (OUR) decreased when ammonium oxidation was about to finish, and was observed as a sudden rise of DO. The slope change of the DO signal was easily recognized (Figure 2). The aeration was automatically stopped when over a period of one minute the slope increased significantly. This aeration phase length control resulted in an average ammonium concentration in the effluent of 1.67 ± 0.57 mgN·L^−1^. Slope changes occurred during the third period of 20 min of full aeration in Phase II and III, and during the sixth aeration pulse of 12.5 min in Phase V with intermittent aeration.

### 3.2. N-removal and NOB Abundance in the Parent Reactor

**Figure 3:**
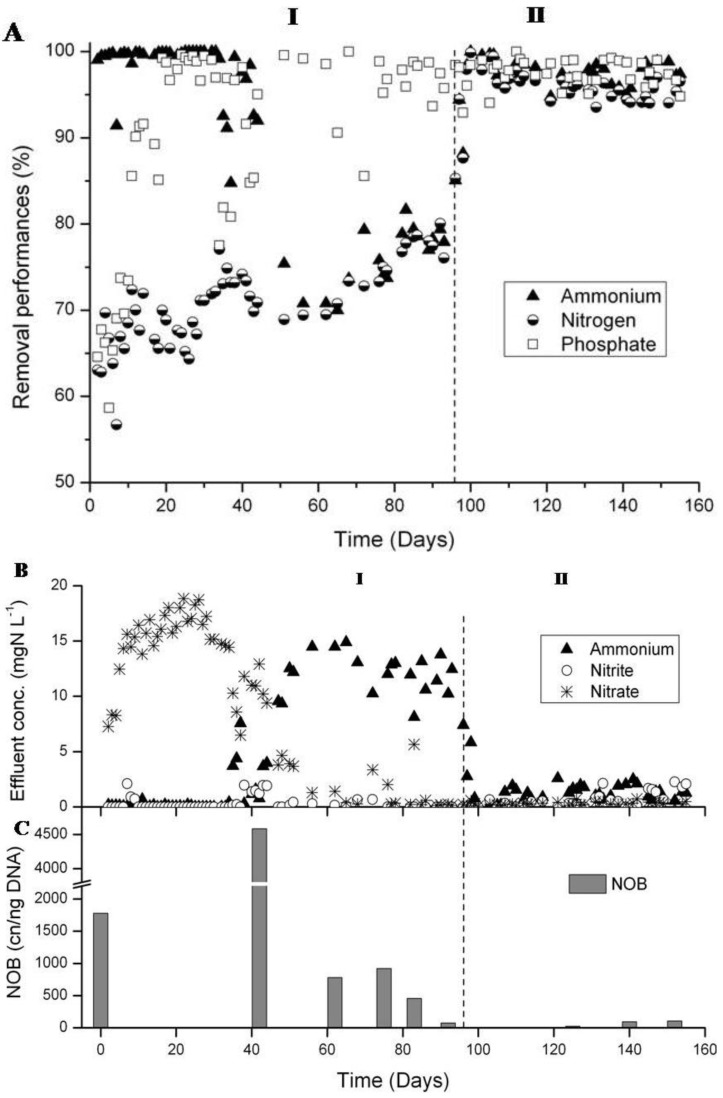
Nutrient removal performances of parent reactor between days 1 and 155. (**A**) Nutrient removal performances. (**B**) Concentrations of N compounds in the effluent and (**C**) *Nitrospira* 16S rRNA gene concentrations measured by qPCR. At day 96 (start of Phase II), the oxygen supply was increased, but automatically stopped upon completion of ammonium oxidation.

During the first 30 days of reactor operation nitrification was close to 100% and the N-removal between 60 and 75% (Figure 3A), High nitrate concentrations of up to 19 mg·L^−1^ were measured in the effluent (Figure 3B). Between day 35 and 50, the nitrification efficiency gradually decreased to 75%. The sludge concentration decreased from 7.5 gTSS·L^−1^ at day 24 to 2.8 gTSS·L^−1^ at day 44 (results shown in [35]). At the same time the sludge transformed from a mixture of flocculent sludge and small granules to a completely granular sludge with a low sludge volume index of 30 mL·g^−1^. After day 44 the sludge concentration gradually recovered to reach 15.3 gTSS·L^−1^ at day 98. After day 100 granular sludge was purged regularly to maintain the concentration between 15 and 17 gTSS·L^−1^, resulting in a sludge retention time of 20–25 days. Nitrification did not recover concurrently with the re-increase of the sludge concentration. Before increasing the set points of the high DO phases on day 81, nitrification and N-removal performance were around 75%, and P-removal was at 97% (Figure 3A). The increase of the set points from 50%, 40% and 30% to 60%, 50% and 40%, respectively, resulted in an increase of NH_4_^+^-removal to 79% without leading to significant NO_x_^−^ accumulation. In Phase II, with full aeration during the high DO phases, nitrification further increased. Between days 100 and 155, NH_4_^+^-removal was at 98%. The NO_x_^−^ effluent concentrations remained very low (Figure 3B) leading to an average N-removal of over 95% for this phase (Figure 3A). 

**Figure 4:**
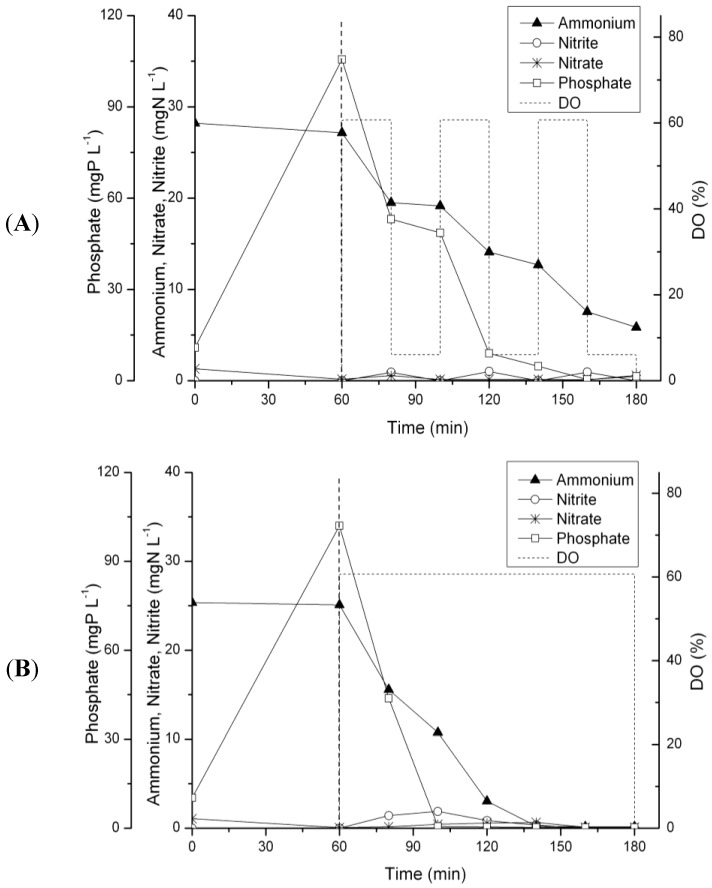
Concentrations of N and P compounds during one SBR cycle (at day 98) operated (**A**) with high-low DO strategy and (**B**) in a test cycle with uncontrolled full aeration. Concentrations at time 0 were calculated based on the effluent concentrations of the previous cycle and the influent concentrations. Aeration started after 60 min of plug-flow feeding (vertical dashed line). The dotted lines show schematically the aeration strategy.

An analysis of the pollutant concentrations during one SBR cycle at day 98 revealed that almost no NO_x_^−^ accumulated during the periods with high DO (Figure 4A). The same result was observed in a test cycle with uncontrolled full aeration. No nitrate and only traces of nitrite were detected (Figure 4B). All ammonium was removed by simultaneous nitrification and denitrification.

A clear decrease of the NOB population was observed after day 100. In the inoculation sludge the concentration of *Nitrospira* 16S rRNA genes was about 1800 cn·ng^−1^ of DNA (Figure 3C). At day 42 until which a clear accumulation of nitrate occurred, this concentration was more than twice as high, but decreased afterwards to values below 1000 cn·ng^−1^ of DNA, and finally, after day 100, the concentration was in the order of 100 cn·ng^−1^ of DNA (Figure 3C).

**Figure 5:**
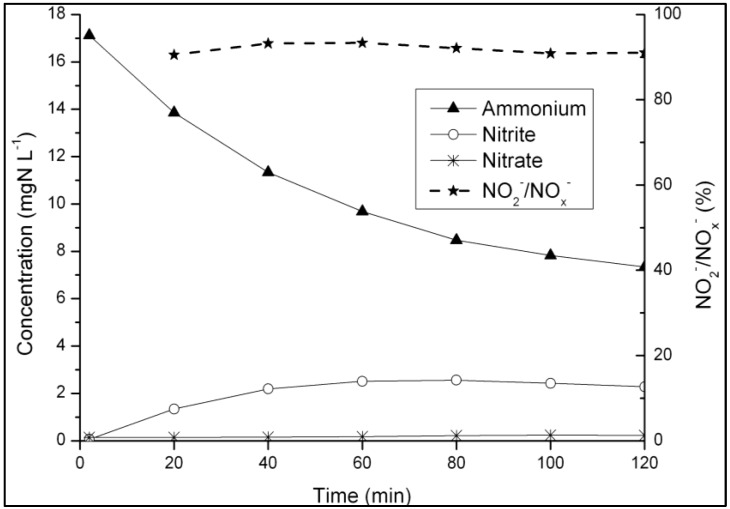
Nitrification batch test with granular sludge taken at the end of the starvation phase without COD supply (at day 118).

Finally, a nitrification batch test with unfed granular sludge was carried out at day 118 to elucidate whether nitrate or nitrite was the main product of ammonium oxidation under unfavorable conditions for denitrification. Between 90% and 94% of the accumulated NO_x_^−^ in the liquid phase was in the form of nitrite while nitrate was hardly detected (Figure 5) which indicated that N-removal occurred mainly over nitrite at that stage of the study.

### 3.3. N-Removal and NOB Abundance at 20 °C

**Figure 6:**
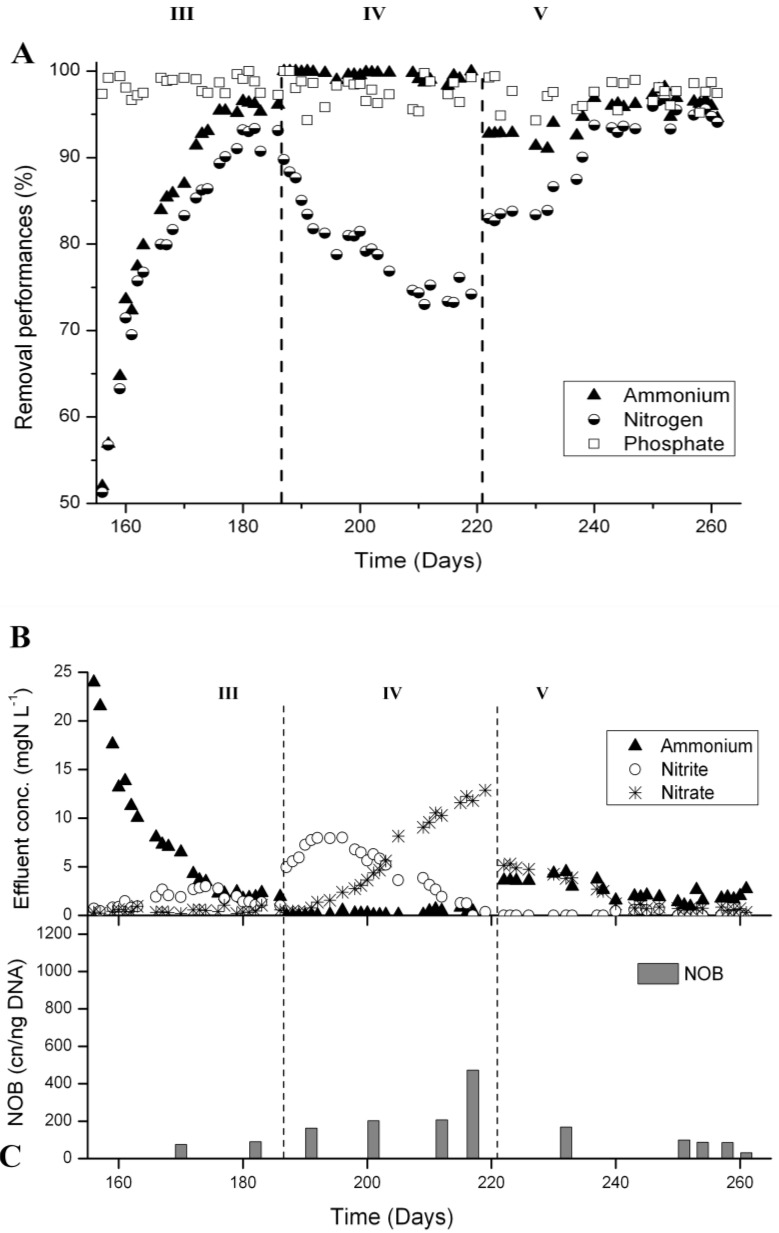
Nutrient removal performances at 20 °C (reactor A) from days 156 to 261. (**A**) Nutrient removal performances. (**B**) Concentrations of N compounds in the effluent and (**C**) *Nitrospira* 16S rRNA gene concentrations measured by qPCR. Until day 186, the reactor was operated with alternate high-low DO and aeration phase length control (Phase III), from days 187 to 220 the reactor was fully aerated during 2 h (Phase IV), and finally, from days 221 to 261 (Phase V), the reactor was operated with intermittent aeration and aeration phase length control.

In the reactor operated at 20 °C (reactor A), 52% of NH_4_^+^-removal was observed on the first day after sludge separation (day 156) (Figure 6A). This sharp performance decrease compared with the parent reactor was due to reduced sludge concentration, from about 16 g·L^−1^ in the parent reactor down to 8 g·L^−1^. During 3 weeks no granular sludge was purged until the sludge concentration reached again 15 g·L^−1^. Nitrification recovered rapidly during Phase III and after one week NH_4_^+^-removal was back at 80%. Between day 180 and 186, NH_4_^+^-removal was on average over 96% and aeration was stopped by automatic aeration phase length control slightly before the end of the normal starvation time of 2 h. The average N-removal during this last week of Phase II was 93%. P-removal was constantly over 97% (Figure 6A). The accumulated NO_x_^−^ was in the form of nitrite (Figure 6B), and the *Nitrospira* abundance remained very low (Figure 6C), indicating N-removal over nitrite.

In Phase IV, after switching to 2 h of full aeration, the N-removal performance gradually decreased from 93% to 74% (Figure 6A). At first, nitrite was the main ammonium oxidation product, but over time more and more nitrate was produced. From day 203 until the end of Phase IV (day 219) nitrate was the main NO_x_^−^ in the effluent (Figure 6B). At the same time, an increase in *Nitrospira* population was observed (Figure 6C).

Finally, in Phase V, the reactor was operated with intermittent aeration and aeration phase length control. N-removal improved immediately to >80%, and after 20 days even to >90%. Between days 241 and 261, the average N-removal was 95% and P-removal 98% (Figure 6A). The nitrate concentration in the effluent decreased to values below 1 mgN·L^−1^ (Figure 6B) and the *Nitrospira* 16S rRNA gene concentrations were below 100 cn·ng^−1^ of DNA at the end of Phase V (Figure 6C). Despite the low NOB concentration observed, no nitrite was detected in the effluent (Figure 6B).

### 3.4. N-Removal and NOB Abundance at 15 °C

**Figure 7:**
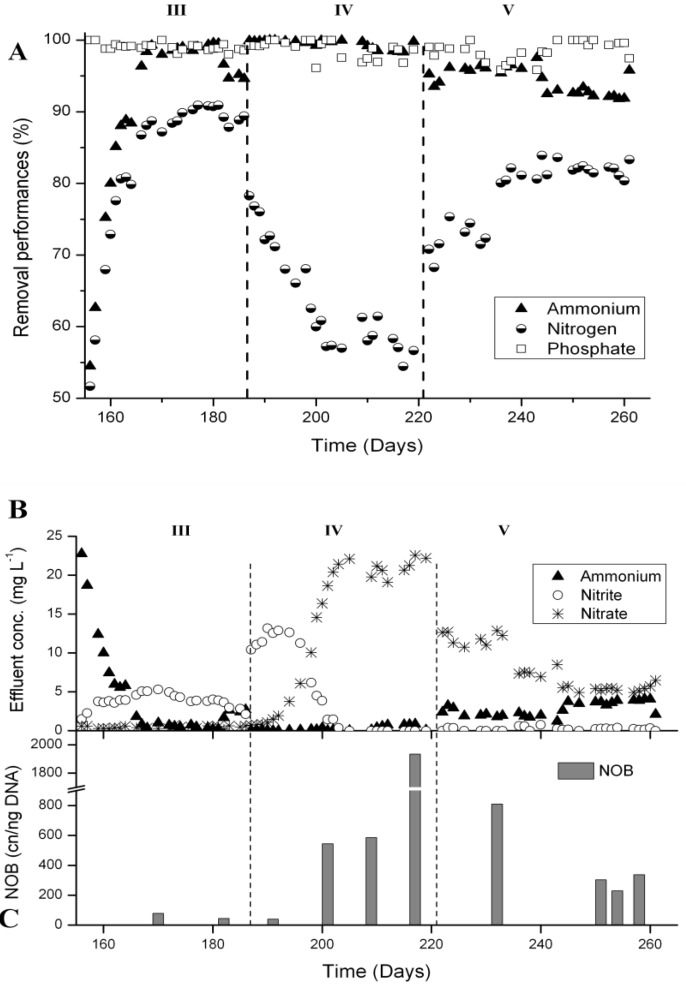
Nutrient removal performances at 15 °C (reactor B) from days 156 to 261. (**A**) Nutrient removal performances. (**B**) Concentrations of N compounds in the effluent and (**C**) *Nitrospira* 16S rRNA gene concentrations measured by qPCR. Until day 186, the reactor was operated with alternate high-low DO and aeration phase length control, from days 187 to 220 the reactor was fully aerated during 2 h, and finally, from days 221 to 261, the reactor was operated with intermittent aeration and aeration phase length control.

At 15 °C (reactor B), NH_4_^+^-removal also decreased to around 50% after sludge separation (day 156), but recovered within two weeks (Figure 7A). The sludge concentration recovered similarly to reactor A in about 3 weeks from 8 g·L^−1^ after sludge separation to 15 g·L^−1^. N-removal stabilized at around 90% and nitrite was the major N compound in the effluent during Phase III (Figure 7B). In Phase IV with to 2 h of full aeration, N-removal immediately decreased to 80% and within two weeks to 55%–60% (Figure 7A). While during the first 10 days mainly nitrite was measured in the effluent, a shift towards total nitrification was observed. After 16 days, no more nitrite was present in the effluent, but about 20 mgN·L^−1^ of nitrate (Figure 7B). The *Nitrospira* 16S rRNA gene concentration increased to almost 2000 cn·ng^−1^ of DNA (Figure 7C). After switching to intermittent aeration and aeration phase length control in Phase V, N-removal increased to about 80% within 17 days (Figure 7A). Concentrations of 4 mgN·L^−1^ of ammonium and 5 mgN·L^−1^ of nitrate were measured in the effluent, but no nitrite (Figure 7B). The concentration of *Nitrospira* 16S rRNA genes clearly decreased, but remained between 200 and 400 cn·ng^−1^ of DNA (Figure 7C).

### 3.5. Nitrous Oxide Emissions and Concentrations in the Liquid Phase

**Figure 8:**
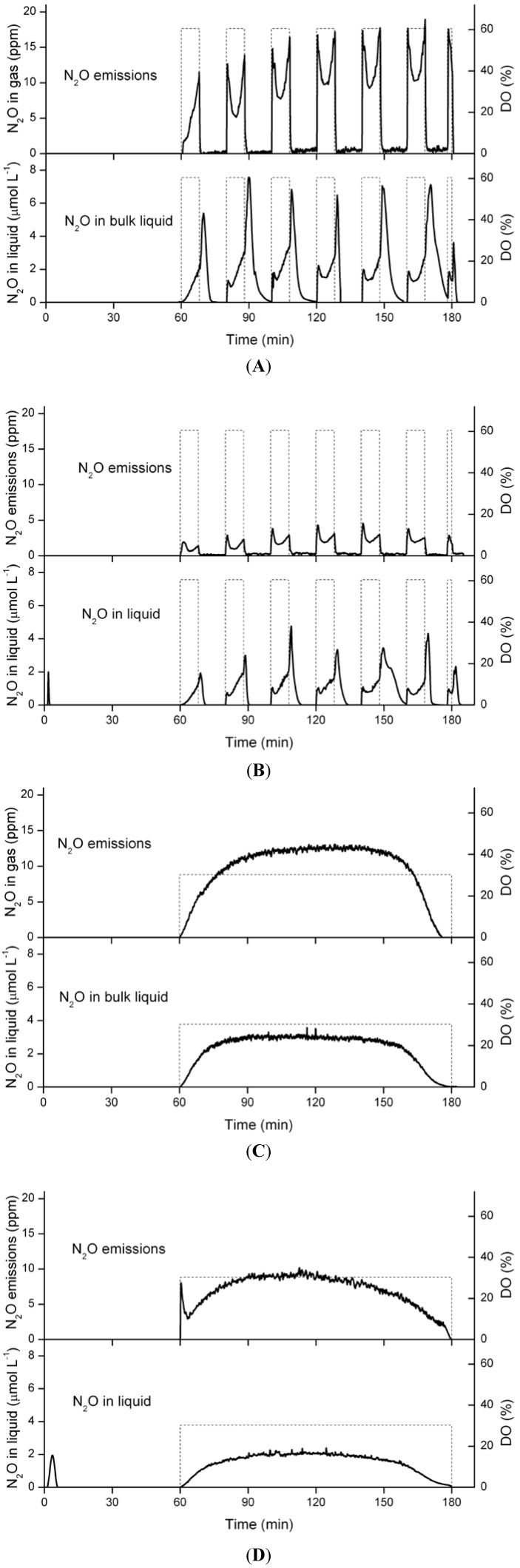
Typical profiles of nitrous oxide concentrations in the effluent gas and the bulk liquid during a SBR cycle. The dashed lines show schematically the aeration strategy: (**A**) N-removal over nitrite with intermittent aeration, (**B**) N-removal over nitrate with intermittent aeration, (**C**) N-removal over nitrite with constant DO of 30% and (**D**) N-removal over nitrate with constant DO of 30%. The shown profiles have been measured with 400 mgCOD·L^−1^ influent concentration.

Nitrous oxide emissions were studied to get an estimation of the impact of N-removal over nitrite, as well as intermittent aeration. With N-removal over nitrite and intermittent aeration, N_2_O was only emitted during the aeration pulses (Figure 8A). However, in the bulk liquid the concentration sharply increased right at the end of the aeration phase (Figure 8A). Except for the first aeration pulse, two peaks of N_2_O emissions were measured during each aeration pulse, one at the beginning and one at the end (Figure 8A). Similar profiles for N_2_O emissions and liquid concentrations were observed with N-removal over nitrate (Figure 8B). However, the concentrations were lower than for N-removal over nitrite. Moreover, with N-removal over nitrate a small peak of N_2_O appeared in the liquid phase during the first minutes of the feeding phase and a N_2_O emission peak was observed at the beginning of the first aeration pulse (Figure 8B,D). 

With a constant DO of 30% and N-removal over nitrite, N_2_O concentrations in the gas and the liquid phase constantly increased during the first minutes of the aeration, before reaching a steady state (Figure 8C). With N-removal over nitrate, the N_2_O concentrations were again a bit lower (Figure 8D). Also with constant DO a peak of N_2_O in the liquid phase was observed during the first minutes of the feeding phase and in the gas phase right after the aeration start (Figure 8D). With N-removal over nitrite, these peaks did not appear.

The N_2_O emissions varied between 0.7% and 12.9% of the influent N load under the different conditions tested (Table 2). The highest N_2_O emission of 12.9% was observed with constant DO, N-removal over nitrite and influent COD concentration of 400 mgCOD·L^−1^, whereas the lowest emission of 0.7% was found with N-removal over nitrate and intermittent aeration. The *Nitrospira* concentration was 22 ± 9 cn·ng^−1^ of DNA in the nitrite pathway sludge, and 445 ± 79 cn·ng^−1^ of DNA in the nitrate pathway sludge. Increased COD concentrations had a decreasing effect on nitrous oxide emissions under almost all conditions tested.

**Table 2:** N_2_O emissions with N-removal over nitrite and nitrate, respectively, with intermittent aeration and constant DO of 30%.

Aeration Strategy	COD Concentration (mgCOD·L^−1^)	Nitrite Pathway (% of N load) ^1^	Nitrate Pathway (% of N load) ^1^
Intermittent aeration	400	5.2 ± 1.1	0.8 ± 0.2
Intermittent aeration	600	2.4 ± 0.8	0.7 ± 0.3
Constant aeration	400	12.9 ± 2.1	9.3 ± 2.4
Constant aeration	600	8.1 ± 1.7	5.9 ± 0.9

^1^ Part of nitrogen load leaving the reactor as N_2_O gas.

## 4. Discussion

Aeration by alternate high-low DO and incomplete nitrification between days 40 and 96 led to a switch from N-removal over nitrate to N-removal over nitrite. Quantitative PCR showed a decrease of the abundance of *Nitrospira* in the granular sludge by over 95% during this period. The subsequent increase of oxygen supply (Phase II) led to N-removal of over 95%, which corresponds to a total N-removal rate of 0.18 kgN m^−3^·d^−1^. Nitrogen was removed from the reactor system in three forms. A previous study with the same wastewater showed that about 25% of N was assimilated into the biomass [35], about 5% leave the reactor as N_2_O gas, and about 65% are denitrified to N_2_. The main denitrifiers within aerobic granular sludge are glycogen- and polyphosphate-accumulating organisms [42], because they are able to store carbon intracellularly during the anoxic feeding phase. In a previous study with the same aeration strategy, the same reactor set-up and the same COD:N:P ratios of 20:2.5:1, it has been observed that COD availability was the limiting factor for denitrification with N-removal over nitrate, resulting in a much lower N-removal of 71% [9]. The very high N-removal of 95% and the low NO_x_^−^ concentration in the effluent indicates that COD is no longer limiting during phase II of the present study. These results agreed with the theory on important COD savings with the nitrite pathway [12] and emphasize the great potential of N-removal over nitrite in COD-limited systems. 

It is not completely clear why nitrification did not recover between day 44 and day 80, despite the sludge concentration re-increase. A possible explanation could be mass transfer limitations of oxygen to granular sludge as previously suggested by Weissbrodt *et al.* [43]. Granular sludge results in a smaller specific surface area than flocculent sludge. However, the incomplete nitrification between days 40 and 96 very likely played a major role in the switch to the nitrite pathway. Ammonium residual has been considered essential to achieve N-removal over nitrite [44,45]. Ammonium residuals are also reported in recent studies investigating partial nitrification for anammox under mainstream conditions [30,31], which is not really surprising since a nitrite-to-ammonium ratio of about 1:1 is targeted in partial nitrification /anammox. The effect of an incomplete nitrification is comparable to an aeration phase length control [25,26]. In those studies, the transition from total to partial nitrification by aeration phase length control has been described as a slow process taking 100–300 days at 20 °C. In our study, the low DO periods of three times 20 min at around 5% presumably accelerated the transition. A DO < 10% has been successfully defined to achieve N-removal over nitrite with granular sludge [22]. Inhibition by free ammonia was very unlikely to play a major role. With 50 mgN-NH_4_^+^·L^−1^ in the influent and a pH controlled between 7.0 and 7.3, the free ammonia concentration remained below 0.4 mgN·L^−1^. For the selective elimination of NOB, free ammonia concentrations of 1–20 mgN·L^−1^ have been reported to be required [46,47]. Inhibition of NOB by free nitrous acid could also be excluded, since no nitrite accumulated during this phase of the study.

Alternation of high-low DO periods and aeration phase length control was successful in maintaining the nitrite pathway with both tested temperatures of 20 °C and 15 °C (Phase III). It confirmed that the alternation of high-low DO periods and aeration phase length control was an efficient strategy to prevent the recovery of NOB, even at 15 °C. It agrees with findings of Yang *et al.* [33], who implemented real-time controlled intermittent aeration based on the pH signal to achieve N-removal over nitrite. They could maintain the nitrite pathway in a SBR while decreasing the temperatures from 22 to 12 °C. 

In the subsequent period with uncontrolled high aeration, full nitrification recovered much faster at 15 °C than at 20 °C (Phase IV). An explanation for this phenomenon could be the higher nitrite accumulation at 15 °C which provided more substrate for the growth of NOB. At 15 °C, nitrate concentration in the effluent reached a steady state after about 15 days. At 20 °C, it gradually increased during the 33 days of uncontrolled full aeration without reaching a steady state. Presumably some N was still removed over nitrite at 20 °C at the end of Phase IV. 

With intermittent aeration and aeration phase length control (Phase V), the nitrite pathway was rapidly recovered at 20 °C. The clear decrease of the *Nitrospira* population and the stable N-removal of 95% indicated that after 20 days N was mainly removed over nitrite, even if some residual nitrate was still observed in the effluent. Several reasons may explain the rapid recovery of the nitrite pathway. First, intermittent aeration was supposed to act similarly as alternate high-low DO on NOB growth rate, but more efficiently. After each pulse, some nitrite would remain and be denitrified in the subsequent non-aerated phase instead of being oxidized by NOB. Hence, the impact on the growth rate should be multiplied by the number of pulses compared to a simple aeration phase length control. A crucial point was to ensure that nitrite was completely removed before the new aeration pulse started. For this reason, relatively long non-aerated phases of 12.5 min between the aeration pulses were defined. Fux *et al.* [28] have implemented an automatic aeration control for intermittent aeration based on the oxidation-reduction potential (ORP) to make sure that all nitrite was denitrified before restarting aeration. Moreover, it has been reported in several studies that transient anoxia favored the out-selection of NOB [27,29,30]. The NOB activity is disturbed by anoxic conditions and recovers only slowly when re-exposed to oxygen [48]. A last reason for the rapid recovery of the nitrite pathway may be that the NOB population was still relatively low at 20°C at the end of Phase IV. At 15 °C, the *Nitrospira* population also decreased with intermittent aeration, but remained 3–4 times higher than at 20 °C. N-removal reached a steady state at around 80%, with mainly nitrate in the effluent. This indicated that at 15 °C intermittent aeration was not able to completely suppress nitrite oxidation. The most likely reason for this is the higher maximum growth rate of NOB compared to AOB at this temperature [14]. 

Over the whole study, only little nitrite accumulated with N-removal over nitrite. The highest concentration measured in the fully aerated reactor during the SBR cycle at day 99 was 1.8 mgN·L^−1^. During the full aeration period (Phase III), the effluent NO_x_^−^ concentration was at both temperatures about 50% lower with N-removal over nitrite than with N-removal over nitrate. With intermittent aeration, no nitrite was detected during the SBR cycle (Supplementary Figure S2A,B), despite of evidence for N-removal over nitrite, at least at 20 °C. The low nitrite concentrations proved that simultaneous nitrification and denitrification was more efficient by the nitrite pathway than the nitrate pathway. In a previous study, 70%–100% higher denitrification rates have been observed for nitrite compared to nitrate [9]. Hence, this result was expected. However, a recent study by Bassin *et al.* [49] with aerobic granular sludge and similar operating conditions reported significantly higher denitrification rates with nitrate than with nitrite. The observation of enhanced denitrifiaction over nitrite should probably be considered to be sludge specific. The accumulation of nitrite is known to have a negative impact on the phosphate uptake [50]. Since nitrite did not accumulate in this study, it was always possible to maintain high P-removal performances in all different phases of the reactor operation.

N_2_O emissions were higher with N-removal over nitrite than over nitrate under all four tested conditions. This agrees with two other recent studies on N_2_O emissions [51,52]. From a mass balance perspective this does not compromise the advantages of N-removal over nitrite compared to N-removal over nitrate, since the difference in N_2_O emissions were only in the order of 2%–5%. Moreover, N_2_O emissions were lower with intermittent aeration than with a constant DO of 30% and overall the applied strategy with intermittent aeration and N-removal over nitrite resulted in lower N_2_O emissions than classical N-removal over nitrate with constant aeration. Low DO concentrations enhance the production of N_2_O [53,54]. With intermittent aeration the DO was either high or oxygen was completely absent. The reason for the N_2_O peak right after aeration stop was most likely the remaining oxygen during the first 1–2 min after aeration stop. SND could still occur, but the N_2_O produced by these processes was not stripped anymore by the aeration. This N_2_O was only removed from the reactor at the beginning of the subsequent aeration pulse, explaining the emission peak right after the start of aeration. With N-removal over nitrate, a N_2_O peak was observed during the first minutes of the feeding phase. This peak was most likely due to heterotrophic denitrification, and the emission peak due to the stripping of this N_2_O. With N-removal over nitrite, almost no nitrite remained at the end of the SBR cycle and therefore no heterotrophic denitrification took place during the subsequent feeding phase. 

The DO signal appeared to be a valid and reliable criterion for the aeration phase length control. It agrees with results of a study by Yang *et al.* [33], where the aeration phase length was also controlled based on the slope of DO. In other studies, the online control of aeration and/or feeding was done based on the pH [26] or the ORP [28]. In the present study, the main reason for choosing a DO-based aeration phase length control rather than a pH-based control was that the pH was regulated during the aerated phases. The drawback of the DO-based control was that the length of the non-aerated phases in intermittent aeration could not be controlled online, since DO gives no indication on denitrification. The pH signal was neither usable to determine the moment when all nitrite was denitrified. A pH drop could be observed during the non-aerated phases. However, the signal was not reliable enough, probably because the bulk liquid was unmixed during the non-aerated periods.

## 5. Conclusions

This study showed that aeration phase length control combined with intermittent aeration or alternate high-low DO, is an efficient way to achieve N-removal over nitrite, which is especially interesting for COD-limited systems. N-removal efficiencies of up to 95% were achieved with this way of reactor operation. At 20 °C, N-removal over nitrite was achieved within 20–60 days and it was possible to switch from N-removal over nitrite to N-removal over nitrate and back again. At 15 °C, the NOB population could be reduced, but nitrite oxidation could not be completely suppressed. However, the combination of aeration phase length control and high-low DO was successful to maintain the nitrite pathway at 15 °C despite the clearly higher maximum growth rate of NOB compared to AOB. 

## Supplementary Files

Supplementary File 1 Supplementary Information (PDF, 451 KB)
