# Supplementary material for: Nitrogen Removal over Nitrite by Aeration Control in Aerobic Granular Sludge Sequencing Batch Reactors
Source: Int J Environ Res Public Health. 2014 Jul 8;11(7):6955–78. doi: 10.3390/ijerph110706955 (PMC4113855; doi:10.3390/ijerph110706955)
Supplement: Supplementary File 1 — Supplementary Information (PDF, 451 KB) [file ijerph-11-06955-s001.pdf]

OPEN ACCESS

International Journal of
Environmental Research and Public Health
ISSN 1660-4601
www.mdpi.com/journal/ijerph

Supplementary Information

Nitrogen Removal over Nitrite by Aeration Control in Aerobic Granular Sludge Sequencing Batch Reactors. *Int. J. Environ. Res. Public Health* 2014, *11*

Samuel Lochmatter, Julien Maillard and Christof Holliger *

Laboratory for Environmental Biotechnology, School of Architecture, Civil and Environmental Engineering, Ecole Polytechnique Fédérale de Lausanne (EPFL), 1015 Lausanne, Switzerland; E-Mails: samuel.lochmatter@epfl.ch (S.L.); julien.maillard@epfl.ch (J.M.)

* To whom correspondence should be addressed; E-Mail: christof.holliger@epfl.ch; Tel.: +41-21-693-47-24; Fax: +41-21-693-47-22.

Table S1. Parameters for individual quantitative PCR runs. B and M are the parameters of the linear calibration curve, B is the constant and M the slope.

Parameters	Run 1	Run 2	Run 3	Run 4	Run 5	Run 6	Run 7
B	33.289	33.735	36.637	37.055	37.379	31.605	35.612
M	−3.537	−3.438	−3.446	−3.422	−3.542	−3.434	−3.514
Efficiency	0.917	0.954	0.951	0.960	0.916	0.955	0.926
R^2	0.999	0.998	0.998	0.994	0.997	0.997	0.998

Table S2. *Nitrospira* 16S rRNA gene concentrations analyzed by qPCR under different aeration conditions during the study period of 261 days.

Reactor	Aeration Strategy	TempErature	Day	*Nitrospira* (cn ng^{-1} of DNA)
	alternating high-low DO	20 °C	0	1779
Parent reactor	alternating high-low DO	20 °C	42	4583
	alternating high-low DO	20 °C	62	781
	alternating high-low DO	20 °C	75	919
	alternating high-low DO	20 °C	83	458
	alternating high-low DO	20 °C	92	76
	alternating high-low DO	20 °C	125	26
	alternating high-low DO	20 °C	140	95
	alternating high-low DO	20 °C	152	107
Reactor 1	alternating high-low DO	15 °C	170	78
	alternating high-low DO	15 °C	182	45
	constant high DO	15 °C	191	40

Table S2. *Cont.*

Reactor	Aeration Strategy	TempErature	Day	*Nitrospira* (cn ng^{-1} of DNA)
	constant high DO	15 °C	201	545
	constant high DO	15 °C	212	586
	constant high DO	15 °C	217	1936
	intermittent aeration	15 °C	232	810
	intermittent aeration	15 °C	251	303
	intermittent aeration	15 °C	254	230
	intermittent aeration	15 °C	258	337
Reactor 2	alternating high-low DO	20 °C	170	75
	alternating high-low DO	20 °C	182	91
	constant high DO	20 °C	191	163
	constant high DO	20 °C	201	203
	constant high DO	20 °C	212	207
	constant high DO	20 °C	217	473
	intermittent aeration	20 °C	232	168
	intermittent aeration	20 °C	251	99
	intermittent aeration	20 °C	254	87
	intermittent aeration	20 °C	258	86

Figure S1. Comparison between gas chromatography and microsensor measurements for nitrous oxide in the gas phase.

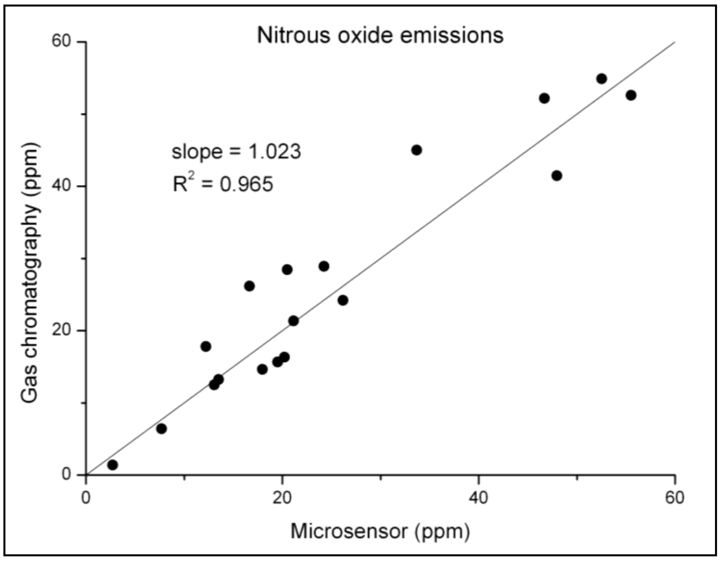

Figure S2. Nutrient removal during SBR cycles operated at day 258 for reactor **A** (20 °C) and reactor **B** (15 °C) with intermittent aeration. Nutrient concentrations at time 0 were calculated based on the effluent concentrations of the previous cycle and the influent concentrations. Aeration started after 60 min of plug-flow feeding (indicated by the vertical dashed line). The dotted lines show schematically the aeration strategy.

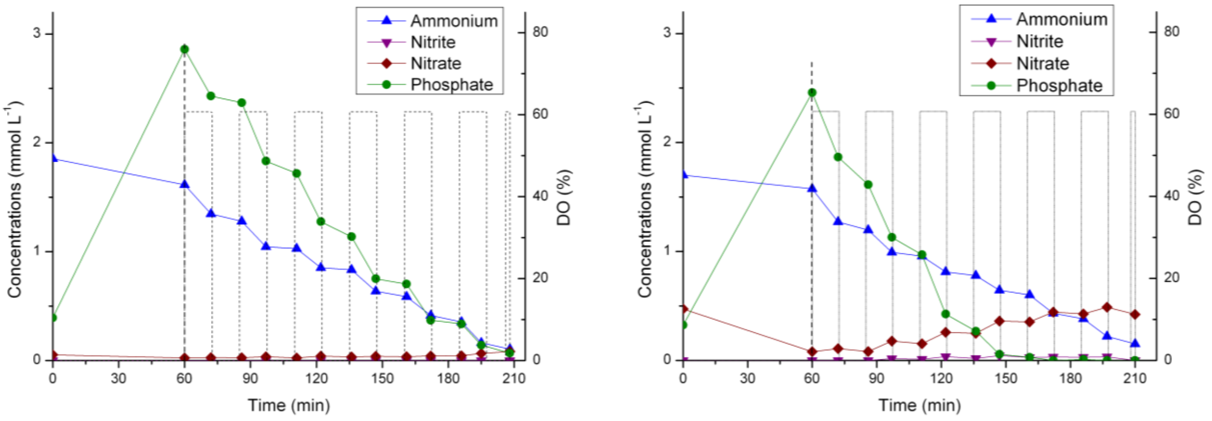

(**A**) (**B**)

© 2014 by the authors; licensee MDPI, Basel, Switzerland. This article is an open access article distributed under the terms and conditions of the Creative Commons Attribution license (http://creativecommons.org/licenses/by/3.0/).
